# Combined mRNA expression levels of members of the urokinase plasminogen activator (uPA) system correlate with disease-associated survival of soft-tissue sarcoma patients

**DOI:** 10.1186/1471-2407-11-273

**Published:** 2011-06-25

**Authors:** Matthias Kotzsch, Viktor Magdolen, Thomas Greither, Matthias Kappler, Matthias Bache, Christine Lautenschläger, Susanne Füssel, Alexander W Eckert, Thomas Luther, Gustavo Baretton, Peter Würl, Helge Taubert

**Affiliations:** 1 Institute of Pathology, Dresden University of Technology, Dresden, Germany; 2 Department of Obstetrics and Gynecology, Technical University of Munich, München, Germany; 3 Center for Reproductive Medicine and Andrology, Martin-Luther-University Halle-Wittenberg, Halle, Germany; 4 Department of Oral and Maxillofacial Plastic Surgery, Martin-Luther-University Halle-Wittenberg, Halle, Germany; 5 Department of Radiotherapy, Martin-Luther-University Halle-Wittenberg, Halle, Germany; 6 Institute of Medical Biometry and Informatics, Martin-Luther-University Halle-Wittenberg, Halle, Germany; 7 Department of Urology, Dresden University of Technology, Dresden, Germany; 8 Medical Laboratory Unit, Bautzen, Germany; 9 Department of General and Oncological Surgery, Clinical Unit Bremen-Mitte, Bremen, Germany; 10 Klinik für Urologie, Universitätsklinikum und Nikolaus-Fiebiger-Zentrum für Molekulare Medizin der Friedrich-Alexander Universität Erlangen-Nürnberg, Erlangen, Germany

## Abstract

**Background:**

Members of the urokinase-type plasminogen activator (uPA) system are up-regulated in various solid malignant tumors. High antigen levels of uPA, its inhibitor PAI-1 and its receptor uPAR have recently been shown to be associated with poor prognosis in soft-tissue sarcoma (STS) patients. However, the mRNA expression of uPA system components has not yet been comprehensively investigated in STS patients.

**Methods:**

The mRNA expression level of uPA, PAI-1, uPAR and an uPAR splice variant, uPAR-del4/5, was analyzed in tumor tissue from 78 STS patients by quantitative PCR.

**Results:**

Elevated mRNA expression levels of PAI-1 and uPAR-del4/5 were significantly associated with clinical parameters such as histological subtype (*P *= 0.037 and *P *< 0.001, respectively) and higher tumor grade (*P *= 0.017 and *P *= 0.003, respectively). In addition, high uPAR-del4/5 mRNA values were significantly related to higher tumor stage of STS patients (*P *= 0.031). On the other hand, mRNA expression of uPA system components was not significantly associated with patients' survival. However, in STS patients with complete tumor resection (R0), high PAI-1 and uPAR-del4/5 mRNA levels were associated with a distinctly increased risk of tumor-related death (RR = 6.55, *P *= 0.054 and RR = 6.00, *P *= 0.088, respectively). Strikingly, R0 patients with both high PAI-1 and uPAR-del4/5 mRNA expression levels showed a significant, 19-fold increased risk of tumor-related death (*P *= 0.044) compared to the low expression group.

**Conclusion:**

Our results suggest that PAI-1 and uPAR-del4/5 mRNA levels may add prognostic information in STS patients with R0 status and distinguish a subgroup of R0 patients with low PAI-1 and/or low uPAR-del4/5 values who have a better outcome compared to patients with high marker levels.

## Background

Soft-tissue sarcomas (STS) are malignant mesenchymal neoplasias with an incidence of about 1% among all human malignancies [1]. STS enlarge leading to the appearance of a pseudo capsule composed of an inner compression zone and an outer reactive zone at formation of fingers, which give rise to satellite lesions several centimeters away from the primary tumor [2]. The major clinical problems in the treatment of STS are the propensity of the tumor to recur locally, and the fact that many patients without obvious clinical metastases harbor occult micro-metastases that become clinically evident. Lymph node metastases are rare in STS patients [3,4]. Despite adequate local control of the primary tumor, about 50% of sarcoma patients will succumb to distant metastatic disease [5].

The urokinase plasminogen activator (uPA) system has been shown to play a major role in the pericellular network of interacting proteolytic systems that are able to degrade extracellular matrix components and facilitate tumor invasion and metastasis [6,7]. Furthermore, components of the uPA system have been implicated in proliferation, migration and adhesion of tumor cells as well as in tumor angiogenesis [8,9]. Components of the uPA system, which consists of the serine protease uPA, its receptor uPAR and its principal inhibitor PAI-1 are prognostic factors in different types of cancer. High antigen levels of uPA and/or PAI-1 protein in tumor tissue extracts are strong predictors of poor prognosis in patients afflicted with different types of solid malignant tumors including sarcomas [10,11]. High uPAR levels are also associated with poor prognosis in various cancer types, however, the prognostic impact of uPAR expression is not as pronounced as that of uPA and PAI-1 [12,13]. In contrast, the expression of a mRNA splice variant of wild-type uPAR (uPAR-wt) lacking exons 4 and 5 (uPAR-del4/5) has been demonstrated to be a highly sensitive, independent prognostic marker in breast cancer patients [14-16].

Whereas wild-type uPAR consists of three structurally homologous domains, in the uPAR-del4/5 variant the complete domain II of uPAR is deleted, and the uPAR-del4/5 protein does not interact with either of its ligands uPA or vitronectin [17]. However, in breast cancer cells the overexpression of the uPAR-del4/5 protein profoundly affects the *in vitro *invasion capacity of cells through a Matrigel matrix, the adhesion to extracellular matrix proteins and also lung colonization in an *in vivo *metastasis model. These observations strongly suggest that uPAR-del4/5 displays biological activity modulating tumor biological relevant processes [17].

In sarcomas, high expression of uPA and uPAR antigen as detected by immunohistochemistry has been reported to be an independent prognostic factor for metastasis-free survival and overall survival in chondrosarcoma patients [18]. In soft-tissue sarcomas, increased uPA protein levels in tumor tissue were found to significantly correlate with local recurrence and metastasis in a cohort of 69 STS patients [19]. We recently reported a highly significant correlation between high antigen levels of uPA, PAI-1 or uPAR in tumor tissue, and of soluble uPAR in serum, with poor outcome of STS patients [20]. However, only one report has investigated the mRNA expression of components of the uPA system in tumor tissue from soft-tissue sarcoma patients [21]. In this study, which included 38 STS patients, significantly higher uPA, PAI-1 and uPAR mRNA expression levels were observed in tumor tissue compared to paired adjacent normal tissue. Additionally, PAI-1 mRNA expression was found to be 6-fold higher in metastatic tumors than in non-metastatic tumors [21].

In the present study, we determined the mRNA expression levels of uPA, PAI-1 and uPAR in a cohort of 78 adult STS patients and analyzed their relationship with prognostically relevant clinical and histomorphological parameters and disease-associated survival. Furthermore, the impact of mRNA expression of uPA system components on disease-associated survival was evaluated in clinically relevant subgroups of STS patients with complete (R0) or non-radical (R1) tumor resection.

## Methods

### Patients and tumor material

For the present study, tumor tissue samples of 78 adult patients with histologically verified soft-tissue sarcomas that have been described in previous studies were used [20,22]. The study adhered to national regulations on ethical issues and was approved by the Ethics Committee from the Medical Faculty of the Martin-Luther-University Halle-Wittenberg, Germany. All patients gave written informed consent (Institute of Pathology, University of Halle-Wittenberg, Germany and Department of Surgery 1, University of Leipzig, Germany). The median age of the patients at surgery was 59 years (range 22-83 years). The median follow-up time of the patients was 42 months (range 2 to 146 months after primary tumor resection). During that time, 30 patients experienced a locoregional recurrence, seven developed a distant metastasis and 42 of the patients have died. The tumors were staged according to the UICC system. All of the tumor samples were collected before radio- or chemotherapy. Relevant data on the clinical and histomorphological parameters of the STS patients are shown in Table 1 and in Additional file 1 .

**Table 1:** Relationship between clinical and histomorphological parameters, and mRNA levels of tumor biological markers in tumor tissue of STS patients

Patients' characteristics	No. cases	uPA	PAI-1	No. cases^a^	uPAR-wt	uPAR-del4/5
		low/high	low/high		low/high	low/high
Total	78			77/76		
Sex^b^		*P *= 0.071	*P *= 0.111		*P *= 0.031	*P *= 0.201
Male	35	21/14	20/15	34/33	20/14	18/15
Female	43	18/25	18/25	43	18/25	20/23
Histological subtype^c, d^		*P *= 0.135	*P *= 0.037		*P *= 0.044	*P *< 0.001
LS	18	10/8	9/9	18	13/5	15/3
MFH	17	6/11	8/9	16/15	6/10	7/8
FS	3	1/2	0/3	3	1/2	1/2
RMS	6	4/2	3/3	6	3/3	1/5
LMS	17	6/11	6/11	17	6/11	4/13
NS	7	5/2	5/2	7	5/2	5/2
Syn	10	7/3	8/2	10	4/6	5/5
Tumor grade^c^		*P *= 0.149	*P *= 0.017		*P *= 0.178	*P *= 0.003
I	15	7/8	6/9	15	8/7	9/6
II	32	20/12	21/11	32	17/15	19/13
III	31	12/19	12/19	30/29	13/17	10/19
Tumor stage^c^		*P *= 0.431	*P *= 0.111		*P *= 0.483	*P *= 0.031
I	13	6/7	5/8	13	7/6	8/5
II	31	19/12	20/11	31	16/15	18/13
III	25	9/16	10/15	24/23	11/13	9/14
IV	9	5/4	4/5	9	4/5	3/6
Complete resection^b^		*P *= 0.056	*P *= 0.304		*P *= 0.437	*P *= 0.200
Radical (R0)	52	22/30	24/28	52/51	23/29	24/27
Not radical (R1)	26	17/9	15/11	25	15/10	14/11
Tumor localization^c^		*P *= 0.197	*P *= 0.071		*P *= 0.098	*P *= 0.394
Extremities	49	22/27	22/27	48/47	19/29	21/26
Trunk wall	6	4/2	3/3	6	5/1	3/3
Head/neck	2	2/0	2/0	2	2/0	1/1
Abdomen/retroperitoneum	21	11/10	12/9	21	12/9	13/8
Patient follow-up^b^		*P *= 0.405	*P *= 0.645		*P *= 0.748	*P *= 0.688
Alive	36	15/21	18/18	36/35	18/18	18/17
Dead	42	24/18	21/21	41	20/21	20/21

^a^The number of cases for uPAR-wt and uPAR-del4/5 is 77 and 76, respectively.

^b^*P *for Mann-Whitney U test, ^c^*P *for Kruskal-Wallis test.

^d^Abbreviations: LS - liposarcoma, MFH - malignant fibrous histiocytoma, FS - fibrosarcoma, RMS - rhabdomyosarcoma, LMS - leiomyosarcoma, NS - neurogenic sarcoma, Syn - synovial sarcoma; the median mRNA values of the uPAR system components in the different histological STS subtypes are presented in Additional file 3.

### Quantification of uPA, PAI-1, uPAR-wt and uPAR-del4/5 mRNA by real-time PCR

Tissue processing, isolation of total RNA, cDNA synthesis, and evaluation of the quantity and quality of the isolated RNA were performed as described [22]. Among the 78 STS patients, the mRNA expression in tumor tissue could be investigated in all cases for uPA and PAI-1 expression, in 77 cases for expression of wild-type uPAR (uPAR-wt) and in 76 cases for expression of the uPAR splice variant uPAR-del4/5.

For quantification of uPA, PAI-1, uPAR-wt and uPAR-del4/5 mRNA, gene-specific hybridization probes and highly sensitive RT-PCR assays (LC FastStart DNA Master Hybridization Probes, Roche Diagnostics, Mannheim, Germany) applying LightCycler technology (Light Cycler 1.0; Software Version 3.5, Roche) were used as previously described [14,23]. Briefly, the PCR assays were performed using 2 μl of a 1:10 dilution of the respective cDNA products. Primer sequences, concentration of primers and probes as well as PCR conditions for amplification are shown in Additional file 2. All HPLC-purified primers were purchased from TibMolBiol (Berlin, Germany). Five-log-range calibration curves were generated for each PCR run using eight glass capillaries coated with defined numbers of linearized plasmid molecules (10^1 ^- 10^6 ^molecules), carrying either the uPA, the PAI-1 or the uPAR (wild-type or -del4/5) cDNA (Roboscreen, Leipzig, Germany). All measurements were performed using aliquots of the same cDNA dilutions within short time intervals to ensure comparable conditions. The cDNA samples were quantified at least in duplicate in independent PCR runs for the appropriate marker transcripts. The mean values of all measurements were used for further calculations. The levels of specific mRNAs were evaluated relative to the average expression levels of the medium abundance housekeeping gene hypoxanthine guanine phosphoribosyltransferase (HPRT) as described [24]. Relative mRNA expression ratios (amol of appropriate marker per amol HPRT) were used for all further calculations and statistical analyses.

### Statistical analysis

The levels of significance between continuous variables of the biological markers were calculated with Spearman's rank correlation test (r_s_). The relationship between biological marker expression levels and clinical and histomorphological parameters was evaluated using non-parametric Mann-Whitney or Kruskal-Wallis tests. For survival analyses the disease-associated survival of STS patients was used as the follow-up end point. The disease-associated survival was defined as the time from the day of primary surgery to tumor-related death of the patients. Statistical analyses of the association between mRNA expression of uPA system components and prognosis were performed by Kaplan-Meier analyses (log-rank tests) and using the Cox proportional hazard regression model. The multivariate Cox regression model was adjusted for the following known clinical prognostic factors in STS patients: tumor stage, histological subtype, type of tumor resection, and tumor localization. All calculations were performed using the SPSS 17.0 program (SPSS-Science, Chicago, IL). *P*-values < 0.05 were considered statistically significant.

## Results

### Expression of uPA, PAI-1, uPAR-wt and uPAR-del4/5 mRNA and their association with clinical and histomorphological parameters

The expression of uPA and PAI-1 mRNA was determined in 78 cases, and the expression of uPAR-wt and uPAR-del4/5 mRNA was determined in 77 and 76 STS samples, respectively (Table 1). The normalized mRNA expression values of uPA, PAI-1, uPAR-wt and uPAR-del4/5 ranged from 0-16.2 (median 0.23; 25th and 75th percentile 0.055 and 1.03, respectively), from 0-181.9 (median 0.73; 25th and 75th percentile 0.10 and 3.08, respectively), from 0-24.1 (median 0.18; 25th and 75th percentile 0.044 and 1.15, respectively), and from 0-0.29 (median 0.004; 25th and 75th percentile 0.0001 and 0.012, respectively), respectively. For statistical analysis, the median value was set as the cut-off point to separate STS patients in groups with low or high tissue mRNA expression.

First, the correlation between the mRNA expression levels of the biological markers was analyzed using Spearman's rank correlation. In general, all factors displayed a strong correlation with each other with r_s_-values of approximately 0.80; uPA mRNA values significantly correlated with PAI-1 (r_s _= 0.82, *P *< 0.001), with uPAR-wt (r_s _= 0.81, *P *< 0.001) and with uPAR-del4/5 (r_s _= 0.74, *P *< 0.001) mRNA levels. In addition, significant correlations were found between uPAR-wt mRNA levels and both PAI-1 and uPAR-del4/5 mRNA values (r_s _= 0.85, *P *< 0.001, and r_s _= 0.82, *P *< 0.001, respectively) as well as between PAI-1 and uPAR-del4/5 mRNA values (r_s _= 0.78, *P *< 0.001).

The relationship between the mRNA expression levels and the clinical and histomorphological parameters of the STS patients is summarized in Table 1. A significant association was observed between mRNA expression levels of PAI-1 (*P *= 0.037), uPAR-wt (*P *= 0.044) and uPAR-del4/5 (*P *< 0.001) and the histological subtype of STS patients. High mRNA expression levels of these three markers (and of uPA) were observed, especially in leiomyosarcomas. Furthermore, mRNA levels of PAI-1 (*P *= 0.017) and uPAR-del4/5 (*P *= 0.003) were significantly related to tumor grade, and high uPAR-del4/5 mRNA values were associated with a higher tumor stage in STS patients (*P *= 0.031). In contrast, mRNA levels of uPA did not differ significantly between tumors in relation to clinical and histomorphological parameters (Table 1).

### Association of uPA, PAI-1, uPAR-wt and uPAR-del4/5 expression with disease-associated survival

The association of clinical and histomorphological parameters and tumor biological factors with disease-associated survival is presented in Table 2. In univariate Cox regression hazard analysis, two of the clinical parameters, tumor stage and tumor resection status were significantly related to prognosis, i.e. patients with high stage tumors or with tumors that were not completely resected (R1) had, as expected, a higher risk for tumor-related death (Table 2). No significant association between uPA, PAI-1, uPAR-wt and uPAR-del4/5 mRNA values with disease-associated survival of STS patients was observed in both univariate (Table 2) and multivariate Cox regression analyses (data not shown).

**Table 2:** Univariate Cox regression analysis for the association of relevant clinical and histomorphological parameters, and of uPA, PAI-1, uPAR-wt and uPAR-del4/5 mRNA levels in tumor tissue of STS patients with disease-associated survival

Factor	No. cases^a^	RR (95% CI)^b^	*P*
			
Histological subtype^c^			
LS	18	1	
MFH/ FS	20	2.35 (0.9-6.2)	0.085
RMS/ LMS	23	2.62 (1.0-6.8)	0.050
NS	7	1.57 (0.4-6.3)	0.522
Syn	10	1.29 (0.4-4.4)	0.686
Tumor stage			
I	13	1	
II	31	2.88 (0.6-12.9)	0.165
III	25	9.27 (2.1-40.6)	0.003
IV	9	33.5 (7.0-161.0)	<0.001
Complete resection			
Radical (R0)	52	1	
Not radical (R1)	26	2.59 (1.4-4.8)	0.002
Tumor localization			
Extremities	49	1	
Trunk wall	6	0.88 (0.2-3.8)	0.858
Head/neck	2	3.79 (0.9-16.7)	0.079
Abdomen/retroperitoneum	21	1.93 (1.0-3.8)	0.057
uPA mRNA^d^			
Low	39	1	
High	39	0.73 (0.4-1.4)	0.319
PAI-1 mRNA^d^			
Low	39	1	
High	39	1.18 (0.6-2.2)	0.609
uPAR-wt mRNA^d^			
Low	39	1	
High	38	1.11 (0.6-2.1)	0.736
uPAR-del4/5 mRNA^d^			
Low	38	1	
High	38	1.06 (0.6-2.0)	0.839

^a^Total number of cases: n = 78, the number of cases for uPAR-wt and uPAR-del4/5 is 77 and 76, respectively.

^b^RR: hazard ratio (95% confidence interval) of univariate Cox regression analysis.

^c^Abbreviations: LS - liposarcoma, MFH - malignant fibrous histiocytoma, FS - fibrosarcoma, RMS - rhabdomyosarcoma, LMS - leiomyosarcoma, NS - neurogenic sarcoma, Syn - synovial sarcoma.

^d^Relative mRNA expression ratio of the respective marker / HPRT dichotomized in high and low levels by median values.

We next performed analyses in the subgroup of STS patients with complete tumor resection (R0) who were at lower risk for tumor-related death compared to the patients whose tumors were not completely resected (R1, Table 2; [25]). We assessed whether some of the biological markers might add prognostic information for patients' survival in the R0 subgroup of STS patients. In R0 patients, survival time is inversely significantly correlated with expression of PAI-1 mRNA (r_s _= -0.337, *P *= 0.014) and of uPAR-del4/5 mRNA (r_s _= -0.346, *P *= 0.013) by bivariate correlation analysis using Spearman's Rho test (Additional file 3: A). Furthermore, using linear regression analysis about a third of patients with lower expression rates for both markers showed the best survival (Additional file 3: B). Therefore it was implicated to apply the 33% percentiles as cut-off points for R0 patients according to their mRNA expression levels, i.e. for PAI-1 (33% percentile: 0.228) and for uPAR-del4/5 mRNA expression (33% percentile: 0.001). Accordingly, R0 patients (n = 52) with mRNA values in the range of the 0% to the 33% percentile were allocated to the low expressing group, and all other patients with mRNA values above the 33% percentile (>33% to 100%) were designated as the high expressing group. We found an association between high PAI-1 and uPAR-del4/5 mRNA expression levels and an increased risk of tumor-related death for R0 patients using multivariate Cox regression analysis with relative risks (RR) of 6.55 (95%CI = 1.0-44.5) and 6.00 (95%CI = 0.8-47.1), respectively, with a trend towards significance (*P *= 0.054 and *P *= 0.088, respectively; Table 3). A moderately increased risk of tumor-related death in R0 patients was also observed for high uPA and uPAR-wt mRNA values (RR = 3.53, 95%CI = 0.7-16.8, and RR = 2.66, 95%CI = 0.5-14.4, respectively). However, this risk was not significantly different than for R0 patients expressing low uPA or uPAR-wt mRNA levels (Table 3).

**Table 3:** Multivariate Cox regression analysis for the association of relevant clinical and histomorphological parameters, and of uPA, PAI-1, uPAR-wt and uPAR-del4/5 mRNA levels with disease-associated survival in the subgroup of STS patients with complete tumor resection (R0)

Factor	No. cases^a^	RR (95% CI)^b^	*P*
			
Histological subtype^c^			
LS	15	1	
MFH/ FS	11	0.76 (0.1-8.0)	0.820
RMS/ LMS	15	0.37 (0.1-3.3)	0.381
NS	4	7.79 (0.2-240.1)	0.240
Syn	6	5.30 (0.4-76.4)	0.219
Tumor stage			
I	12	1	
II	19	0.80 (0.1-17.2)	0.889
III	16	10.97 (0.7-160.8)	0.080
IV	4	347.3 (13.6-8869.8)	<0.001
Tumor localization			
Extremities	35	1	
Trunk wall	6	1.08 (0.1-8.7)	0.939
Head/neck	2	7.44 (0.5-101.8)	0.133
Abdomen/retroperitoneum	8	2.23 (0.3-18.4)	0.454
uPA mRNA^d, e^			
Low	17	1	
High	35	3.53 (0.7-16.8)	0.114
PAI-1 mRNA^d, e^			
Low	17	1	
High	35	6.55 (1.0-44.5)	0.054
uPAR-wt mRNA^d, e^			
Low	17	1	
High	35	2.66 (0.5-14.4)	0.260
uPAR-del4/5 mRNA^d, e^			
Low	17	1	
High	34	6.00 (0.8-47.1)	0.088

^a^Total number of cases: n = 52, the number of cases for uPAR-del4/5 is 51.

^b^RR: hazard ratio (95% confidence interval) of multivariate Cox regression analysis.

^c^Abbreviations: LS - liposarcoma, MFH - malignant fibrous histiocytoma, FS - fibrosarcoma, RMS - rhabdomyosarcoma, LMS - leiomyosarcoma, NS - neurogenic sarcoma, Syn - synovial sarcoma.

^d^Relative mRNA expression ratio of the respective marker / HPRT divided into groups using the 33% percentile; Low: 0 - 33% percentile; High: > 33% - 100% percentile.

^e^Factors were added separately to the base model of relevant clinical prognostic factors in STS patients including the histological subtype, tumor stage and tumor localization.

**Figure 1:**
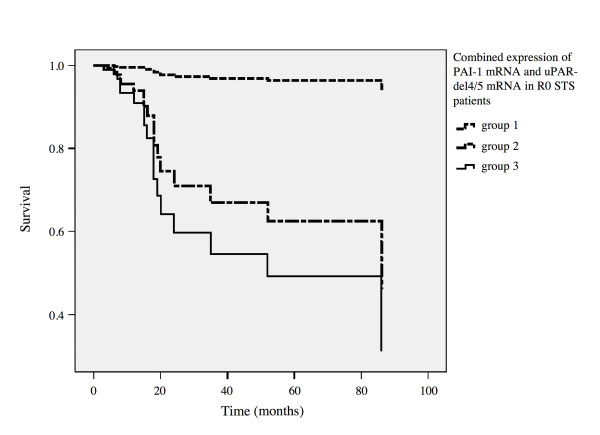
**Multivariate Cox regression analysis for the association of combined PAI-1/uPAR-del4/5 mRNA values with disease-associated survival in the subgroup of STS patients with complete tumor resection (R0)**. For each, the PAI-1 and the uPAR-del4/5 mRNA level, the 33% percentile of the relative expression ratio was used as cut-off point. Group 1 (n = 13), low expression levels of PAI-1/uPAR-del4/5 mRNA; group 2 (n = 8), one of the mRNA values was either low or high; group 3 (n = 30), high expression levels of PAI-1/uPAR-del4/5 mRNA. R0 patients of group 3 (high expression of PAI-1/uPAR-del4/5 mRNA) showed a 19-fold increased risk of tumor-related death (RR = 19.1, 95%CI = 1.1-335.3, *P *= 0.044) compared to group 1 with low expression of PAI-1/uPAR-del4/5 mRNA.

Finally, we assessed whether a combination of PAI-1 and uPAR-del4/5 mRNA values might improve the prognostic impact for patients' survival. Again, the mRNA expression levels of both PAI-1 and uPAR-del4/5 were divided into groups with low (0-33% percentile) *versus *high (> 33% to 100% percentile) values, respectively. In multivariate Cox regression analysis, we found that patients with low mRNA values of both factors were characterized by a much better disease-associated survival than patients with tumors in which one or both mRNA values were high (Figure 1). R0 patients with high PAI-1/uPAR-del4/5 (n = 30) revealed a significant 19-fold increased risk of tumor-related death (RR = 19.1, 95%CI = 1.1-335.3, *P *= 0.044) compared to R0 patients who showed low PAI-1/uPAR-del4/5 mRNA expression levels (n = 13). Thus, a pronounced additive effect on prognosis of R0 patients was identified when combined PAI-1/uPAR-del4/5 mRNA expression levels in tumor tissue were used. The risk of tumor-related death for R0 patients with a low/high mRNA expression (high PAI-1/low uPAR-del4/5 and low PAI-1/high uPAR-del4/5) was not significantly different from that for R0 patients with a low PAI-1/uPAR-del4/5 mRNA expression in their tumors. We did not observe any significant correlation for either group with the disease-associated survival of all STS patients or for the R1 patients. The combination of other factors did also not provide any additional prognostic information.

## Discussion

The uPA system is one of the best-investigated protease systems in both physiological and pathological conditions, including cancer [reviewed in [6,7]]. In clinical studies, high protein contents of uPA and/or PAI-1 in tumor tissue have been shown to indicate an unfavorable prognosis in various types of cancer [10,11,26]. For example, the determination of antigen levels of uPA and PAI-1 in tissue extracts of primary breast cancer has entered clinical practice for risk-adapted, individual therapy decisions in patients with lymph node-negative disease [11,26]. In soft-tissue sarcoma, however, few studies have analyzed the protein expression of uPA system components and evaluated the impact of expression of these proteins on prognosis of STS patients. Choong et al. [19] reported that increasing uPA protein levels in tumor tissue were associated with local recurrence and metastasis in 69 STS patients. Recently, high protein levels of uPA, PAI-1 and uPAR in tumor tissue extracts were found to be significantly associated with a shortened disease-associated survival of STS patients; this association was independent of prognostically relevant clinical parameters [20]. Moreover, combined values of high uPA, PAI-1 or uPAR tissue levels with high serum levels of soluble uPAR were highly significantly and independently associated with poor disease-associated survival; these patients showed an approximately 6-fold increased risk of tumor-related death [20].

Generally, measurements of tumor biological markers in tissue extracts by ELISA require relatively large amounts of fresh-frozen tumor material as well as adequate storage capacities for tumor samples. Furthermore, tumor tissue material is more limited when the cancer is diagnosed at early stages because most tumor tissue specimens are retrieved from fine needle aspirates or core biopsies [27]. Thus, alternative, less material-consuming methods for the quantitative determination of prognostic factors in tumor tissue such as quantitative PCR are desirable.

In the present study, we analyzed the mRNA expression levels of uPA system components in a cohort of 78 patients with soft-tissue sarcoma, and high correlations were found between the mRNA values of uPA, PAI-1, uPAR-wt and an uPAR splice variant, uPAR-del4/5 (with r_s _values greater than 0.70). In agreement with these results, positive correlations between mRNA values of uPA system components have been observed in several other studies [23,28-31]. At the protein level, similar significant correlations between uPA, PAI-1 and uPAR antigen levels in tumor tissue have been reported [20,23,30,32,33]. These findings may not be surprising because various interactions of the members of the plasminogen activation system may affect each other or are regulated in a concerted manner in tumor growth and metastasis.

In contrast, we found only low/moderate correlations between uPA, PAI-1 and uPAR-wt mRNA expression and the respective antigen levels in tumor tissue from the STS patient cohort (r_s _values ranging from 0.26 to 0.35; data not shown). Other studies have also indicated that uPA and/or PAI-1 antigen levels in tumor tissue do not completely reflect the respective mRNA expression level [23,28,30,34,35]. This discrepancy could be due to posttranscriptional regulation processes, which have previously been reported for components of the uPA system [36].

Although mRNA levels do not completely mirror antigen contents in tumor tissue, a number of studies have demonstrated that mRNA expression levels of certain uPA system members are linked with clinical and histomorphological parameters, and/or prognosis in cancer patients. In breast cancer, Witzel et al. [35] have reported that patients with high PAI-1 mRNA expression have a reduced 10-year disease-free survival and overall survival rate, and PAI-1 mRNA expression might reveal additional clinically relevant information compared to PAI-1 protein levels. In other studies that have analyzed PAI-1 mRNA expression in different sets of breast cancer patients, high PAI-1 mRNA levels were found to be significantly associated with shorter metastasis-free or overall survival, whereas uPA mRNA levels had no prognostic relevance [37,38]. However, in a subset of ErbB2-positive breast cancer patients, Urban et al. [29] have identified uPA mRNA as the most significant marker associated with distant metastasis-free survival, whereas PAI-1 mRNA was found to be significantly associated with distant metastasis-free survival, independent of the ErbB2 status. In addition, high uPA and PAI-1 mRNA levels were found to be significantly associated with shorter disease-free survival in primary breast cancer patients, independent of hormone receptor or lymph node status [28]. In other tumor types, such as pancreatic and gastric cancer, mRNA expression of uPA system components has also been found to be related to prognosis [31,39-41]. However, the existence of studies in which no relationship was found between uPA, PAI-1 or uPAR mRNA expression and patients' survival is notable [30,42].

Other studies have also examined uPA system components in soft-tissue sarcomas. In a small cohort of 38 STS patients, significantly higher PAI-1 mRNA expression levels, but not uPA or uPAR levels, were observed in metastatic compared to non-metastatic tumors [21]. In the present study, uPA, PAI-1 and uPAR-wt mRNA values did not differ significantly between tumors in relation to clinical and histomorphological parameters, except for a significant relationship between high PAI-1 mRNA expression and tumor grade and between PAI-1 and uPAR-wt mRNA levels and the histological STS subtype, which has also been reported by others [21]. Interestingly, mRNA levels of uPAR-del4/5, an uPAR mRNA splice variant described recently [14], were significantly correlated with clinical prognostic factors such as histological subtype, tumor grade and tumor stage in STS patients. However, the expression levels of uPAR-del4/5 mRNA, which has recently been shown to be strongly associated with prognosis in lymph node-negative breast cancer patients [16], had no prognostic impact in STS patients. Similarly, mRNA expression of the other uPA system markers did not show a significant correlation with disease-associated survival in the whole cohort of STS patients.

However, when we analyzed the expression pattern of uPA system markers in the subgroup of STS patients who underwent radical tumor resection (R0) we observed a trend towards an association between mRNA expression and disease-associated survival. Specifically, elevated mRNA values of uPAR-del4/5 and PAI-1 were associated with a 6-fold increased risk of tumor-related death in R0 patients (but not in R1 patients). It is tempting to speculate that increasing the number of patients in this subgroup analysis would result in significant associations between high levels of uPAR-del4/5 or PAI-1 mRNA levels and disease-associated survival. Moreover, by using combined PAI-1 and uPAR-del4/5 mRNA expression values, R0-resected patients with high PAI-1/uPAR-del4/5 mRNA values had a significant, 19-fold increased risk of tumor-related death compared to R0-resected patients who displayed low PAI-1/uPAR-del4/5 expression levels. These results suggests that the quantitative determination of PAI-1 and uPAR-del4/5 mRNA expression levels in R0-resected STS patients provides additional prognostic information that may allow for individual, risk-adapted (adjuvant) therapy decisions. Thus, R0 patients at high risk, as identified by elevated uPAR-del4/5 and/or PAI-1 mRNA levels, may benefit from chemotherapy, which would result in the reduction of tumor progression. In contrast, patients displaying low mRNA expression of both uPAR-del4/5 and PAI-1 in tumor tissue could be spared the exposure to chemotherapy.

## Conclusions

In the present study, we analyzed mRNA expression levels of uPA, PAI-1, uPAR and an uPAR mRNA splice variant, uPAR-del4/5, in a cohort of 78 adult soft-tissue sarcoma patients. Our data suggest that mRNA levels of PAI-1 and uPAR-del4/5 are significantly related to clinical and histomorphological parameters of STS patients such as histological subtype, tumor grade or tumor stage. In addition, in STS patients with complete tumor resection high PAI-1 or uPAR-del4/5 mRNA levels are associated with a distinctly shortened disease-associated survival of STS patients. Strikingly, co-detection of a high mRNA level of uPAR-del4/5 and PAI-1 in tumor tissue of STS patients with complete tumor resection is associated with a 19-fold increased risk of tumor-related death suggesting that both markers can be considered together for prognostic evaluation and may support future therapy stratification.

## Competing interests

The authors declare that they have no competing interests.

## Authors' contributions

MK, VM and HT designed the study, collected data, performed statistical analyses and drafted the manuscript. TG, MK, MB, CL, SF, AE, TL, and GB substantially contributed to data acquisition and analysis as well as data interpretation. PW treated the patients, collected the material and data, and reviewed the manuscript. All authors have read and approved the final manuscript version.

## Pre-publication history

The pre-publication history for this paper can be accessed here:

http://www.biomedcentral.com/1471-2407/11/273/prepub

## Supplementary Material

Additional file 1 **Median mRNA values of the uPAR system components in the different histological STS subtypes. **This file contains additional information on the mRNA expression pattern in the seven histological tumor subtypes of the STS patient cohort. Click here for file

Additional file 2 **Sequences of primers and hybridization probes, primer concentrations and amplification conditions for uPA, PAI-1, uPAR-wt and uPAR-del4/5 applied in the RT-PCR LightCycler assay. **This file contains additional information regarding the characteristics of primers and probes (sequence, concentration), and the cycling conditions of the PCR. Click here for file

Additional file 3 **Association of uPAR-del4/5 and PAI-1 mRNA expression levels with disease-associated survival in the subgroup of STS patients without residual tumor mass (R0). **This file contains additional statistical data: **A. **The bivariate correlation between mRNA expression of uPAR-del4/5 and PAI-1 with survival time of R0-STS patients (Spearman's Rho test), and **B**. The association of mRNA expression of uPAR-del4/5 and PAI-1 with survival time of R0-STS patients (Regression model). Click here for file

## References

[B1] Enzinger FM Weiss SW Enzinger FM, Weiss SW General considerations Soft Tissue Tumors 1995 St Louis, Missouri: Mosby 1 16

[B2] Giuliano AE Eilber FR The rationale for planned reoperation after unplanned total excision of soft-tissue sarcoma J Clin Oncol 1985 3 1344 1348 4045526 10.1200/JCO.1985.3.10.1344

[B3] Chang AE Sondak VK Enzinger FM, Weiss SW Clinical evaluation and treatment of soft tissue tumors Soft Tissue Tumors 1995 St Louis, Missouri: Mosby 17 38

[B4] Fong Y Coit DG Woodruff JM Brennan MF Lymph node metastasis from soft tissue sarcoma in adults: Analysis of data from a prospective database of 1772 sarcoma patients Ann Surg 1993 217 72 77 10.1097/00000658-199301000-00012 8424704 PMC1242736

[B5] Potter DA Glenn J Kinsella T Patterns of recurrence in patients with high grade soft-tissue sarcomas J Clin Oncol 1985 3 353 358 3973646 10.1200/JCO.1985.3.3.353

[B6] Duffy MJ McGowan PM Gallagher WM Cancer invasion and metastasis: changing views J Pathol 2008 214 283 293 10.1002/path.2282 18095256

[B7] McMahon B Kwaan HC The plasminogen activator system and cancer Pathophysiol Haemost Thromb 2008 36 184 194 19176991 10.1159/000175156

[B8] Mondino A Blasi F uPA and uPAR in fibrinolysis, immunity and pathology Trends Immunol 2004 25 450 455 10.1016/j.it.2004.06.004 15275645

[B9] Mengele K Napieralski R Magdolen V Reuning U Gkazepis A Sweep F Brünner N Foekens J Harbeck N Schmitt M Characteristics of the level-of-evidence-1 disease forecast cancer biomarkers uPA and its inhibitor PAI-1 Expert Rev Mol Diagn 2010 10 947 962 10.1586/erm.10.73 20964613

[B10] Dass K Ahmad A Azmi AS Sarkar SH Sarkar FH Evolving role of uPA/uPAR system in human cancers Cancer Treat Rev 2008 34 122 136 10.1016/j.ctrv.2007.10.005 18162327

[B11] Schmitt M Mengele K Napieralski R Magdolen V Reuning U Gkazepis A Sweep F Brünner N Foekens J Harbeck N Clinical utility of level-of-evidence-1 disease forecast cancer biomarkers uPA and its inhibitor PAI-1 Expert Rev Mol Diagn 2010 10 1051 1067 10.1586/erm.10.71 21080821

[B12] de Bock CE Wang Y Clinical significance of urokinase-type plasminogen activator receptor (uPAR) expression in cancer Med Res Rev 2004 24 13 39 10.1002/med.10054 14595671

[B13] Hoyer-Hansen G Lund IK Urokinase receptor variants in tissue and body fluids Adv Clin Chem 2007 44 65 102 17682340

[B14] Luther T Kotzsch M Meye A Langerholc T Füssel S Olbrich N Albrecht S Ockert D Muehlenweg B Friedrich K Grosser M Schmitt M Baretton G Magdolen V Identification of a novel urokinase receptor splice variant and its prognostic relevance in breast cancer Thromb Haemost 2003 89 705 717 12669126

[B15] Kotzsch M Farthmann J Meye A Füssel S Baretton G Tjan-Heijnen VCG Schmitt M Luther T Sweep FCGJ Magdolen V Span PN Prognostic relevance of uPAR-del4/5 and TIMP-3 mRNA expression levels in breast cancer Eur J Cancer 2005 41 2760 2768 10.1016/j.ejca.2005.09.002 16256342

[B16] Kotzsch M Sieuwerts AM Grosser M Meye A Füssel S Meijer-van Gelder ME Smid M Schmitt M Baretton G Luther T Magdolen V Foekens JA Urokinase receptor splice variant uPAR-del4/5-associated gene expression in breast cancer: identification of rab31 as an independent prognostic factor Breast Cancer Res Treat 2008 111 229 240 10.1007/s10549-007-9782-6 17952591

[B17] Sato S Kopitz C Grismayer B Beaufort N Reuning U Schmitt M Luther T Kotzsch M Krüger A Magdolen V Overexpression of the urokinase receptor mRNA splice variant uPAR-del4/5 affects tumor-associated processes of breast cancer cells in vitro and in vivo Breast Cancer Res Treat 2011 127 649 657 10.1007/s10549-010-1042-5 20635136

[B18] Morii T Yabe H Morioka H Yamada R Nakagawa T Toyama Y Prognostic relevance of urokinase type plasminogen activator, its receptor and inhibitors in chondrosarcoma Anticancer Res 2000 20 3031 3036 11062719

[B19] Choong PF Fernö M Akerman M Willén H Långström E Gustafson P Alvegård T Rydholm A Urokinase-plasminogen-activator levels and prognosis in 69 soft-tissue sarcomas Int J Cancer 1996 69 268 272 10.1002/(SICI)1097-0215(19960822)69:4<268::AID-IJC5>3.0.CO;2-V 8797866

[B20] Taubert H Würl P Greither T Kappler M Bache M Lautenschläger C Füssel S Meye A Eckert AW Holzhausen HJ Magdolen V Kotzsch M Co-detection of members of the urokinase plasminogen activator system in tumor tissue and in serum correlates with a poor prognosis for soft-tissue sarcoma patients Br J Cancer 2010 102 731 737 10.1038/sj.bjc.6605520 20051950 PMC2837565

[B21] Benassi MS Ponticelli F Azzoni E Gamberi G Pazzaglia L Chiechi A Conti A Spessotto P Scapolan M Pignotti E Bacchini P Picci P Altered expression of urokinase-type plasminogen activator and plasminogen activator inhibitor in high-risk soft tissue sarcomas Histol Histopathol 2007 22 1017 1024 17523079 10.14670/HH-22.1017

[B22] Würl P Kappler M Meye A Bartel F Köhler T Lautenschläger C Bache M Schmidt H Taubert H Co-expression of survivin and TERT and risk of tumor-related death in patients with soft-tissue sarcoma Lancet 2002 359 943 945 10.1016/S0140-6736(02)07990-4 11918915

[B23] Biermann JC Holzscheiter L Kotzsch M Luther T Kiechle-Bahat M Sweep FCGJ Span PN Schmitt M Magdolen V Quantitative RT-PCR assays for the determination of urokinase-type plasminogen activator and plasminogen activator inhibitor type 1 mRNA in primary tumor tissue of breast cancer patients: comparison to antigen quantification by ELISA Int J Mol Medicine 2008 21 251 259 18204793

[B24] Schmidt U Fuessel S Koch R Baretton G Lohse A Tomasetti S Unversucht S Froehner M Wirth MP Meye A Quantitative multi-gene expression profiling of primary prostate cancer Prostate 2006 66 1521 1534 10.1002/pros.20490 16921506

[B25] Lewis JJ Leung D Woodruff JM Brennan MF Retroperitoneal soft-tissue sarcoma: analysis of 500 patients treated and followed at a single institution Ann Surg 1998 228 355 365 10.1097/00000658-199809000-00008 9742918 PMC1191491

[B26] Harbeck N Kates R Gauger K Willems A Kiechle M Magdolen V Schmitt M Urokinase-type plasminogen activator (uPA) and its inhibitor PAI-1: novel tumor-derived factors with a high prognostic and predictive impact in breast cancer Thromb Haemost 2004 91 450 456 14983219 10.1160/TH03-12-0798

[B27] Schmitt M Lienert S Prechtel D Sedlaczek E Welk A Reuning U Magdolen V Jänicke F Sweep CGJ Harbeck N The urokinase protease system as a target for breast cancer prognosis and therapy: Technical considerations J Clinical Ligand Assay 2002 25 43 52

[B28] Spyratos F Bouchet C Tozlu S Labroquere M Vignaud S Becette V Lidereau R Bieche I Prognostic value of uPA, PAI-1 and PAI-2 mRNA expression in primary breast cancer Anticancer Res 2002 22 2997 3003 12530032

[B29] Urban P Vuaroqueaux V Labuhn M Delorenzi M Wirapati P Wight E Senn HJ Benz C Eppenberger U Eppenberger-Castori S Increased expression of urokinase-type plasminogen activator mRNA determines adverse prognosis in ErbB2-positive primary breast cancer J Clin Oncol 2006 24 4245 4253 10.1200/JCO.2005.05.1912 16963728

[B30] Castelló R Landete JM España F Vázquez C Fuster C Almenar SM Ramón LA Radtke KP Estellés A Expression of plasminogen activator inhibitors type 1 and type 3 and urokinase plasminogen activator protein and mRNA in breast cancer Thromb Res 2007 120 753 762 10.1016/j.thromres.2006.12.016 17258797

[B31] Xue A Scarlett CJ Jackson CJ Allen BJ Smith RC Prognostic significance of growth factors and the urokinase-type plasminogen activator system in pancreatic ductal adenocarcinoma Pancreas 2008 36 160 167 10.1097/MPA.0b013e31815750f0 18376307

[B32] Bouchet C Hacène K Martin PM Becette V Tubiana-Hulin M Lasry S Oglobine J Spyratos F Dissemination risk index based on plasminogen activator system components in primary breast cancer J Clin Oncol 1999 17 3048 3057 10506599 10.1200/JCO.1999.17.10.3048

[B33] Foekens JA Peters HA Look MP Portengen H Schmitt M Kramer MD Brünner N Jänicke F Meijer-van Gelder ME Henzen-Logmans SC van Putten WL Klijn JG The urokinase system of plasminogen activation and prognosis in 2780 breast cancer patients Cancer Res 2000 60 636 643 10676647

[B34] Castelló R Estellés A Vázquez C Falcó C España F Almenar SM Fuster C Aznar J Quantitative real-time reverse transcription-PCR assay for urokinase plasminogen activator, plasminogen activator inhibitor type 1, and tissue metalloproteinase inhibitor type 1 gene expressions in primary breast cancer Clin Chem 2002 48 1288 1295 12142386

[B35] Witzel ID Milde-Langosch K Wirtz RM Roth C Ihnen M Mahner S Zu Eulenburg C Jänicke F Müller V Comparison of microarray-based RNA expression with ELISA-based protein determination of HER2, uPA and PAI-1 in tumor tissue of patients with breast cancer and relation to outcome J Cancer Res Clin Oncol 2010 136 1709 1718 10.1007/s00432-010-0829-4 20204407 PMC11828136

[B36] Nagamine Y Medcalf RL Muñoz-Cánoves P Transcriptional and posttranscriptional regulation of the plasminogen activator system Thromb Haemost 2005 93 661 675 15841310 10.1160/TH04-12-0814

[B37] Sternlicht MD Dunning AM Moore DH Pharoah PD Ginzinger DG Chin K Gray JW Waldmann FM Ponder BA Werb Z Prognostic value of PAI-1 in invasive breast cancer: evidence that tumor-specific factors are more important than genetic variation in regulating PAI-1 expression Cancer Epidemiol Biomarkers Prev 2006 15 2107 2114 10.1158/1055-9965.EPI-06-0351 17119035 PMC2731792

[B38] Leissner P Verjat T Bachelot T Paye M Krause A Puisieux A Mougin B Prognostic significance of urokinase plasminogen activator and plasminogen activator inhibitor-1 mRNA expression in lymph node- and hormone receptor-positive breast cancer BMC Cancer 2006 6 216 10.1186/1471-2407-6-216 16945123 PMC1564186

[B39] Lee KH Bae SH Lee JL Hyun MS Kim SH Song SK Kim HS Relationship between urokinase-type plasminogen receptor, interleukin-8 gene expression and clinicopathological features in gastric cancer Oncology 2004 66 210 217 10.1159/000077997 15218312

[B40] Nielsen A Scarlett CJ Samra JS Gill A Li Y Allen BJ Smith RC Significant overexpression of urokinase-type plasminogen activator in pancreatic adenocarcinoma using real-time quantitative reverse transcription polymerase chain reaction J Gastroenterol Hepatol 2005 20 256 263 10.1111/j.1440-1746.2004.03531.x 15683429

[B41] Kita Y Fukagawa T Mimori K Kosaka Y Ishikawa K Aikou T Natsugoe S Sasako M Mori M Expression of uPAR mRNA in peripheral blood is a favourite marker for metastasis in gastric cancer cases Br J Cancer 2009 100 153 159 10.1038/sj.bjc.6604806 19050704 PMC2634681

[B42] Warnecke-Eberz U Prenzel KL Baldus SE Metzger R Dienes HP Bollschweiler E Hoelscher AH Schneider PM Significant down-regulation of the plasminogen activator inhibitor 1 mRNA in pancreatic cancer Pancreas 2008 36 173 177 10.1097/MPA.0b013e31815ac538 18376309

