# Supplementary material for: Combined mRNA expression levels of members of the urokinase plasminogen activator (uPA) system correlate with disease-associated survival of soft-tissue sarcoma patients
Source: BMC Cancer. 2011 Jun 25;11:273. doi: 10.1186/1471-2407-11-273 (PMC3152967; doi:10.1186/1471-2407-11-273)
Supplement: Additional file 2 — Sequences of primers and hybridization probes, primer concentrations and amplification conditions for uPA, PAI-1, uPAR-wt and uPAR-del4/5 applied in the RT-PCR LightCycler assay. This file contains additional information regarding the characteristics of primers and probes (sequence, concentration), and the cycling conditions of the PCR. [file 1471-2407-11-273-S2.DOC]

**Additional file 2.** Sequences of primers and hybridization probes, primer concentrations and amplification conditions for uPA, PAI-1, uPAR-wt and uPAR-del4/5 applied in the RT-PCR LightCycler assay.

|  | | Oligonucleotide sequence (5’- 3’) | Fragment size (bp) |
| --- | --- | --- | --- |
| uPA | Forward Primer (uPA ex10F)  Reverse Primer (uPA ex10,11A)  Hyb Probe 1 (uPA FL)  Hyb Probe 2 (uPA LC) | AGT GTC AGC AGC CCC ACT  CCC CCT GAG TCT CCC TGG  AAG TCA CCA CCA AAA TGC TAT GTG CT  CTG ACC CCC AAT GGA AAA CAG ATT C | 103 |
| PAI-1 | Forward Primer (PAI-1 ex5,6F)  Reverse Primer (PAI-1 ex6,7R)  Hyb Probe 1 (PAI-1 FL)  Hyb Probe 2 (PAI-1 LC) | CTC CTG GTT CTG CCC AAG TT  GAG AGG CTC TTG GTC TGA AAG  TCG GTC ATT CCC AGG TTC TCT AGG  GCT TCC TGA GGT CGA CTT CAG TCT CC | 132 |
| uPAR-wt | Forward Primer (uPAR ex2F)  Reverse Primer (uPAR-wt ex4R)  Hyb Probe 1 (uPAR FL)  Hyb Probe 2 (uPAR LC) | GAC CTC TGC TGC AGG ACC ACG AT  AGG TAA CGG CTC CGG GAA T  GGT ACA GCT TTT ­CTC CAC CAG CTC CA CTC TTC TCC TTC TTC CCA CAA GCG | 196 |
| uPAR-del4/5 | Forward Primer (uPAR ex2F)  Reverse Primer (uPAR-d4/5 ex3,6R)  Hyb Probe 1 (uPAR FL)  Hyb Probe 2 (uPAR LC) | GAC CTC TGC TGC AGG ACC ACG AT  TTT CAA GCT CCA GGA CAG AGT T  GGT ACA GCT TTT ­CTC CAC CAG CTC CA  CTC TTC TCC TTC TTC CCA CAA GCG | 182 |

The specific PCR fragments were amplified as follows:

**uPA and PAI-1** - pre-denaturation 10 min at 95 °C followed by 45 cycles of amplification: denaturation for 10 sec at 95 °C, annealing for 10 sec at 62 °C (uPA) or 63 °C (PAI-1), and elongation for 5 sec at 72 °C. PCR was performed using 0.5 µM of each amplification primer, 0.2 µM of each hybridization probe, and either 5 mM (uPA) or 3.5 mM Mg^2+^ (PAI-1) in a total volume of 20 µl.

**uPAR-wt and uPAR-del4/5** - pre-denaturation 10 min at 95 °C followed by 40 cycles of amplification: denaturation for 10 sec at 95 °C, annealing for 15 sec at 66 °C (uPAR-2/3/4) and 15 sec at 63 °C (uPAR-del4/5), and elongation for 10 sec at 72 °C. Concentration of primers and probes ranged between 0.2 and 0.5 µM, Mg^2+^ was applied at a final concentration of 3 mM and 4 mM for uPAR-wt and uPAR-del4/5, respectively, in a total volume of 20 µl.
