# Supplementary material for: Combined mRNA expression levels of members of the urokinase plasminogen activator (uPA) system correlate with disease-associated survival of soft-tissue sarcoma patients
Source: BMC Cancer. 2011 Jun 25;11:273. doi: 10.1186/1471-2407-11-273 (PMC3152967; doi:10.1186/1471-2407-11-273)
Supplement: Additional file 3 — Association of uPAR-del4/5 and PAI-1 mRNA expression levels with disease-associated survival in the subgroup of STS patients without residual tumor mass (R0). This file contains additional statistical data: A. The bivariate correlation between mRNA expression of uPAR-del4/5 and PAI-1 with survival time of R0-STS patients (Spearman's Rho test), and B. The association of mRNA expression of uPAR-del4/5 and PAI-1 with survival time of R0-STS patients (Regression model). [file 1471-2407-11-273-S3.DOC]

**Additional file 3.** Association of uPAR-del4/5 and PAI-1 mRNA expression levels with disease-associated survival in the subgroup of STS patients without residual tumor mass (R0).

**A**. Spearman’s Rho test: Bivariate correlation between mRNA expression of uPAR-del4/5 and PAI-1 with survival time of R0-STS patients

| Spearman’s-Rho test | | uPAR-del4/5/HPRT | PAI-1/HPRT |
| --- | --- | --- | --- |
| uPAR-del4/5amol/HPRTag/µl | correlation coefficient | 1.000 | .799 |
|  | Sig. (2-sided) | . | 2.19E-12 |
|  | N | 51 | 51 |
| PAI-1amol/HPRTag/µl | correlation coefficient | .799 | 1.000 |
|  | Sig. (2-sided) | 2.19E-12 | . |
|  | N | 51 | 52 |
| survival time | correlation coefficient | -.346 | -.337 |
|  | Sig. (2-sided | .013 | .014 |
|  | N | 51 | 52 |

**B**. Regression model: Association of mRNA expression of uPAR-del4/5 and PAI-1 with survival time of R0-STS patients
